# Class-attention-based lesion proposal convolutional neural network for strawberry diseases identification

**DOI:** 10.3389/fpls.2023.1091600

**Published:** 2023-01-26

**Authors:** Xiaobo Hu, Rujing Wang, Jianming Du, Yimin Hu, Lin Jiao, Taosheng Xu

**Affiliations:** ^1^ Science Island Branch, University of Science and Technology of China, Hefei, Anhui, China; ^2^ Institute of Intelligent Machines, Hefei Institutes of Physical Science, Chinese Academy of Sciences (CAS), Hefei, Anhui, China; ^3^ Institute of Physical Science and Information Technology, Anhui University, Hefei, Anhui, China; ^4^ School of Internet, Anhui University, Hefei, Anhui, China

**Keywords:** convolutional neural network, strawberry disease identification, complex background, similar diseases, class response map, main lesion object, lesion details

## Abstract

Diseases have a great impact on the quality and yield of strawberries, an accurate and timely field disease identification method is urgently needed. However, identifying diseases of strawberries in field is challenging due to the complex background interference and subtle inter-class differences. A feasible method to address the challenges is to segment strawberry lesions from the background and learn fine-grained features of the lesions. Following this idea, we present a novel Class-Attention-based Lesion Proposal Convolutional Neural Network (CALP-CNN), which utilizes a class response map to locate the main lesion object and propose discriminative lesion details. Specifically, the CALP-CNN firstly locates the main lesion object from the complex background through a class object location module (COLM) and then applies a lesion part proposal module (LPPM) to propose the discriminative lesion details. With a cascade architecture, the CALP-CNN can simultaneously address the interference from the complex background and the misclassification of similar diseases. A series of experiments on a self-built dataset of field strawberry diseases is conducted to testify the effectiveness of the proposed CALP-CNN. The classification results of the CALP-CNN are 92.56%, 92.55%, 91.80% and 91.96% on the metrics of accuracy, precision, recall and F1-score, respectively. Compared with six state-of-the-art attention-based fine-grained image recognition methods, the CALP-CNN achieves 6.52% higher (on F1-score) than the sub-optimal baseline MMAL-Net, suggesting that the proposed methods are effective in identifying strawberry diseases in the field.

## 1 Introduction

Strawberry, often praised as the “Queen of Fruits”, is rich in vitamin C and antioxidants that support heart health and blood sugar control (Hannum, 2004). It is becoming a new income-producing agricultural product compared with traditional crops. However, strawberries are very delicate and highly susceptible to infection in natural environment. They are prone to various infectious diseases caused by fungal, bacterial and viral pathogens (Iqbal et al., 2021). Up to now, many strawberry diseases have been identified during the whole cultivation period of strawberries. These diseases can occur in strawberries’ fruit, leaf, and stem, such as gray mold, powdery mildew and anthracnose. Therefore, disease management is a routine and labor-intensive requirement in strawberry cultivation. Currently, the identification of strawberry diseases is empirically conducted by growers, especially in China. The various types of diseases pose a great challenge to the accurate identification of the growers. Meanwhile, the manual manners are expensive, laborious and subjective, making them hard to wildly apply in modern agriculture. Hence, the current strawberry disease management cannot meet the need for automatic monitoring in agricultural practice (Hu et al., 2021). Furthermore, most strawberry growers lack professional knowledge to distinguish the diseases, resulting in the use of incorrect and overdose fungicides in disease management. The abuse of fungicides greatly harms the health of consumers and has caused substantial economic loss (Wang et al., 2021b). There is an urgent need for a fast and effective method to identify diseases in strawberry farming.

In general, the visual symptoms, including color, texture, shape and location of the lesions are important evidence for disease identification (Sankaran et al., 2010; Cruz et al., 2019; Liang et al., 2019). Given these visual features, various methods based on computer vision (CV) technology have been developed to identify different crop diseases. The CV-based methods for crop disease identification can be summarized into two streams. In the first stream, the traditional CV-based methods (such as color space transform, histogram of oriented gradient and gray level co-occurrence matrix [GLCM]) are applied to extract lesion features from diseased spots (Kim et al., 2009; Revathi and Hemalatha, 2014; Kaur et al., 2016; Johannes et al., 2017). Then, a classifier (e.g., linear/logistic regression, random forest) is constructed to yield classification results based on the extracted features (Huang, 2007; Kaur et al., 2016; Iqbal et al., 2018; Dwivedi et al., 2021). For instance, three phalaenopsis seedlings diseases had been successfully identified by an artificial neural network with the GLCM extracted texture features (Huang, 2007). Besides, (Johannes et al., 2017) designed two descriptors of their segmented hot-spot blobs to validate the effectiveness of the related traditional CV-based methods in identifying diseases at the early stage under a complex field background. The two descriptors were used to describe the color and texture features of the blob lab channels, respectively. These studies have proved that traditional CV-based methods are effective in recognizing the diseases of crops in both laboratory and field environments. However, these methods rely on the manual selection of discriminative features among diseases. The discriminative feature selection in field disease identification is very difficult and time-consuming (Zhao et al., 2022). Furthermore, the identification accuracy could dramatically decrease with a slight change in the input image dataset (Arsenovic et al., 2019). These shortcomings result in the traditional CV-based methods rarely adopted in the practice of crop disease identification. The convolutional neural network (CNN) and its variants lead the second stream for crop disease identification. The CNN-based models can automatically extract basic features like color, texture, edge, and location information. Meanwhile, they are competent to obtain more abstract semantic information from the image of crop diseases (Zeiler and Fergus, 2014). Besides, these CNN-based models have more flexible architectures that can be applied as feature extractors or classifiers. In recent studies, the CNN-based models have become the preferred method to identify crop diseases (Liang et al., 2019; Hu et al., 2021; Yang et al., 2022; Zhao et al., 2022). Earlier studies apply the classical CNN models, such as AlexNet (Krizhevsky et al., 2012), GoogLeNet (Szegedy et al., 2015), and ResNet (He et al., 2016) on some specific crop disease datasets and found the most suitable model for the disease identification tasks (Mohanty et al., 2016; Srdjan. et al., 2016; Ferentinos, 2018; Too et al., 2019; Picon et al., 2019). The related models achieve preferable recognition accuracy on their disease datasets. However, these studies fail to consider the complexity of the practical application of field disease identification. The main challenges of field disease identification are the complex background and a variety of diseases with similar symptoms (Barbedo, 2018). These models cannot be applied to crop cultivation practice. Consequently, some researches aim at reducing the misclassification caused by complex backgrounds and diseases with similar symptoms.

A simple yet effective method to eliminate the influence of complex background on disease identification is to segment the lesion region from their background. Several CNN-based semantic segmentation methods have been proposed to mitigate the adverse impact of the background. (Ngugi et al., 2020) proposed a segmentation network, KijianiNet, to segment tomato leaves from the natural field conditions. (Hu et al., 2021) and (Wang et al., 2021a) adopted U-Net (Ronneberger et al., 2015) and DeepLabV3+ (Chen et al., 2018) in the first stage of their models to segment the diseased leaves from the field scenes, respectively. The related experimental results showed that extracting diseased regions from the background can greatly improve the identification performance of the models. However, CNN-based semantic segmentation methods require pixel-level supervision. Such pixel-level annotation by experts is time-consuming, laborious and costly since plenty of lesions have varied shapes. On the topic of similar disease identification, few studies have proposed effective approaches to tackle this issue. (Cruz et al., 2019) applied transfer learning and data augmentation technologies to enhance the ability of the classical CNN models (e.g., AlexNet, GoogLeNet and ResNet) to distinguish the grapevine yellow from its similar diseases (such as grapevine leafroll and stictocephala bisonia). The experimental results confirmed that the data augmentation technologies were beneficial for classical CNN models to identify grape diseases. Because a suitable data augmentation strategy could increase the differences among similar diseases. However, the strategy was not easy to obtain, it required trial and error. The research of (Yang et al., 2022) was a development in identifying similar diseases of field crops. Similar diseases were classified by increasing the weight of discriminative lesion features. To locate lesion details and learn discriminative lesion features among similar diseases, they proposed a self-supervised multi-network fusion classification model. However, the locations of the lesion details were randomly generated. Furthermore, all the obtained lesion details need to be fed to a classifier to assess the confidence of these regions as lesions, which greatly increased the time consumption of the model.

Image-based automatic disease identification is a basic need of modern large-scale cultivation agriculture. Field disease identification is challenging due to the complex background and similar symptoms among diseases. To address these problems, this paper focuses on strawberry field disease identification and proposes a novel Class-Attention-based Lesion Proposal Convolutional Neural Network (CALP-CNN) to precisely identify strawberry diseases in the field. The CALP-CNN method first utilizes a class-attention mechanism to enhance the localization of discriminative lesion feature. Two specific modules (i.e., the class object location module, COLM, and the lesion part proposal module, LPPM) are designed to recursively segment the main lesion object and lesion detail from an input image. Finally, the features of the original, main lesion object and lesion details are concatenated for final identification. To our knowledge, the CALP-CNN method is the first attempt to simultaneously address the challenges posed by the complex background and similar symptoms to crop disease identification in the field. The main contributions are summarized as follows:

- We introduce a new class attention mechanism (i.e., the class response map) to improve the ability of the CNN to localize the discriminative lesion features.
- We address the challenges of field disease identification by developing a novel CALP-CNN that simultaneously removes the noisy background and effectively learns discriminative lesion representations among similar diseases in an unsupervised way.
- A series of experiments are conducted on the field strawberry disease dataset to evaluate the effectiveness of the CALP-CNN. The experimental results show that the proposed method has better performance than other state-of-the-art fine-grained methods on field strawberry disease identification.

## 2 Material and methods

### 2.1 Material

**Figure 1:**
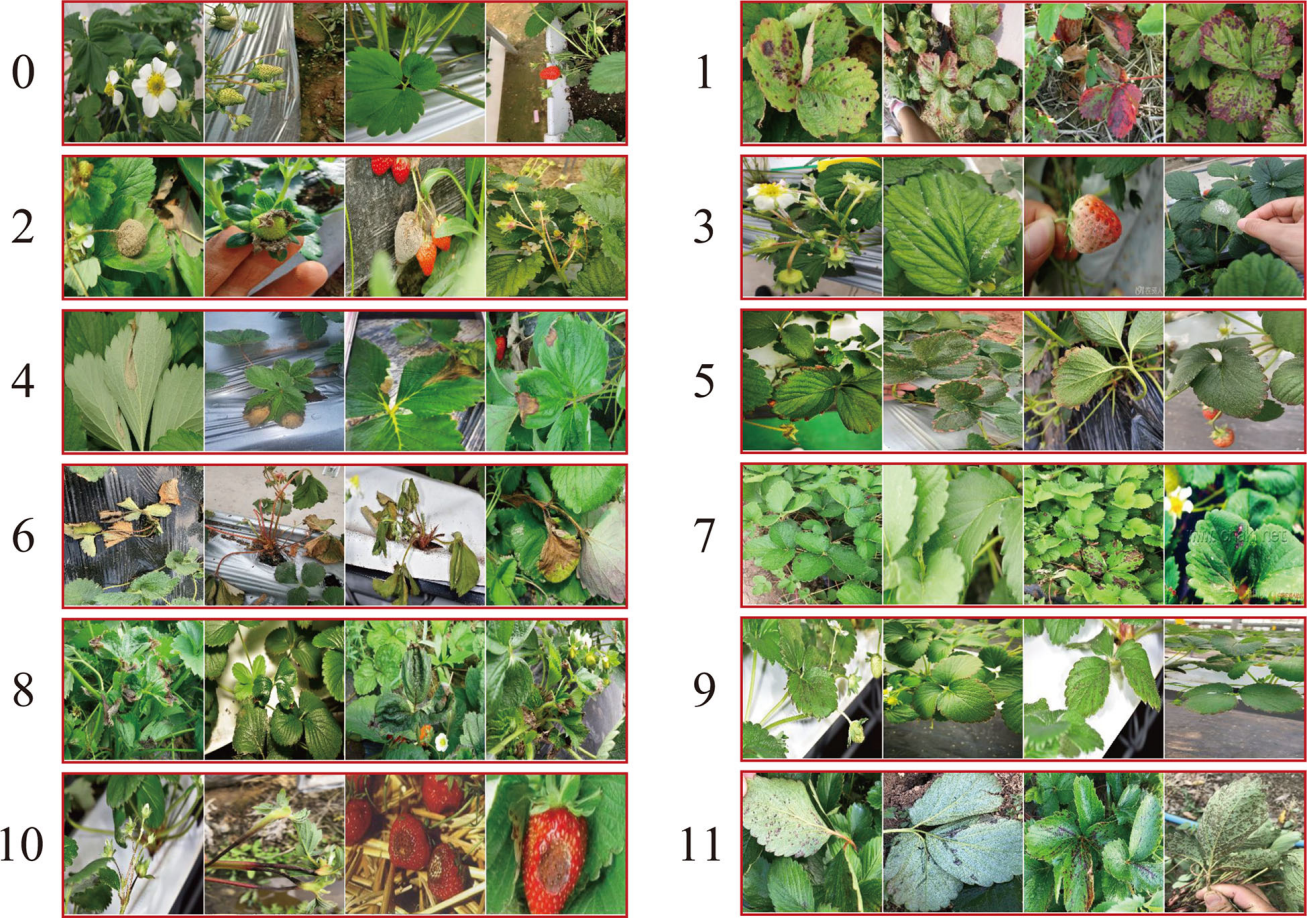
The typical symptoms of 11 common strawberry diseases and one healthy type. The annotated labels of the diseases are one-to-one correspondence with **Table 1**.

In this paper, the strawberry diseases with high incidence in planting practice were taken as our research objects. To this end, a strawberry common disease dataset (SCDD) was constructed. The SCDD was collected in two ways: field-collection and internet crawling. We firstly shot 1,326 disease images of three strawberry varieties (Fengxiang, Nvfeng and Hongyan) in ChangFeng County, Anhui Province, China. To increase the diversity of the dataset, the images were deliberately captured in the field at different angles and focal lengths. The second part was from the internet. A crawler was applied to download more than 5,000 images of field strawberry diseases. The collected images were manually screened one by one to discard the poor-quality samples (obscure and the resolution less than 224×224). The disease images in the dataset were annotated by three experts. One was responsible for labeling the dataset, and the other two were responsible for reviewing the results. Finally, a high-quality dataset of strawberry diseases with 3,411 images was constructed for downstream analysis. The SCDD contained 11 common diseases and healthy type. **Table 1** shows detailed information of the SCDD. In addition, the typical symptoms of 11 common strawberry diseases are shown in **Figure 1**.

**Table 1:** List of strawberry common disease dataset.

Category label	Strawberry disease	Number
0	healthy	509
1	leaf scorch	287
2	gray mold	332
3	powdery mildew	344
4	brown spot	215
5	fertilizer disorder	308
6	fusarium wilt	145
7	white leaf spot	259
8	calcium deficiency	431
9	magnesium deficiency	197
10	anthracnose	198
11	bacterial leaf spot	186
Total		3411

In our experiments, the dataset was randomly divided into a training set, a validation set and a testing set in the ratio of 6:2:2 (2,047 images for training, 682 images for validation, and the remaining 682 images for testing). In the training process, we adopted the online data augmentation strategies to increase the diversity of the dataset and the robustness of the models. Specifically, the processes of Normalize, RandomHorizontalFlip, RandomVerticalFlip, and RandomResizedCrop (crop to 224×224) were applied during training.

### 2.2 Methods

**Figure 2:**
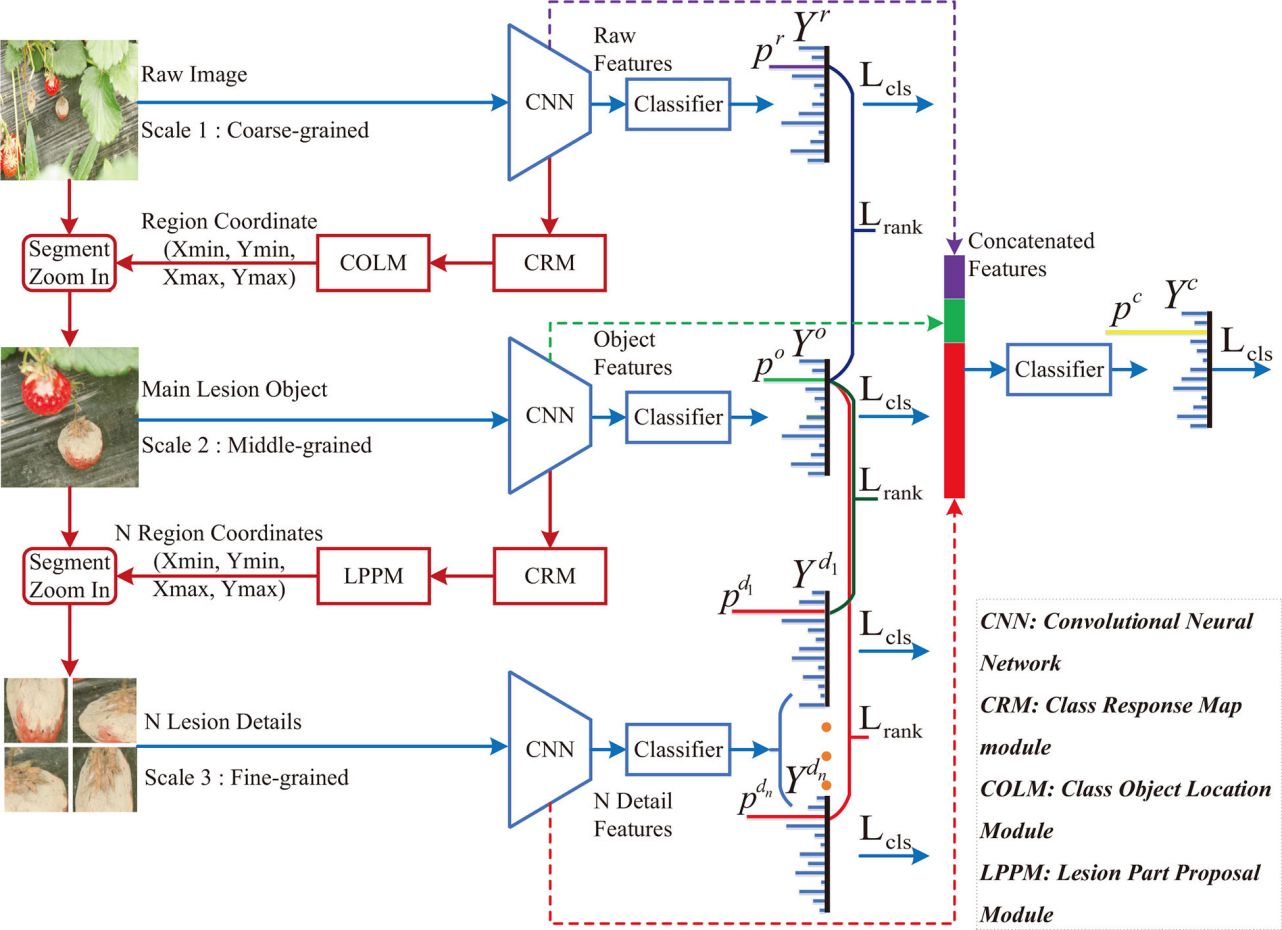
The framework of the proposed CALP-CNN. A cascade architecture is designed to construct the lesion details at different scales. A CNN-based backbone is repeatedly used to extract features from the coarse raw image to lesion detail images. The CRM module generates the class response map from the features. The COLM and the LPPM can obtain the coordinates of the lesion object and the lesion details, respectively. All features (the stripes marked with purple, green, and red) are concatenated for final identification. The classification loss *L_cls_* (cross-entropy loss between ground truth label Y^*^ and predict label *Y^r^*, *Y°*, $Y_{i}^{d}$ ,Y^c^) and the pairwise ranking loss *L_rank_* (the loss between raw probability *p^r^*, object probability *p°*, and lesion probabilities $p^{d_{i}}$ ) are combined to optimize the network and make it converge.

In this paper, a class-attention-based lesion proposal CNN is presented to settle the main challenges of CNN-based methods in field disease identification, i.e., the complex background and similar diseases. The framework of CALP-CNN is shown as **Figure 2**. A cascade architecture is designed for extracting the region-based features from the input images at three scales including the raw image at coarse-grained level, the main lesion object at medium-grained level and the lesion detail images at fine-grained level. Furthermore, a series of modules are developed to extract class-related features in each layer of the cascade architecture. The detailed information of the CALP-CNN is described as follows:

First, a CNN backbone is repeatedly applied to extract region-based features from the input images in three scales. The CNN modules in three scales are given the same parameters. Second, the features are fed forward to three classifiers to predict three probability scores. The computed probability scores represent the prediction confidence of each disease category. Meanwhile, a class response map (CRM) module is constructed to generate a class attention matrix based on the region-based features. Here, the class attention matrix is defined as a class response map in this paper. Third, two different modules (COLM and LPPM) are developed to detect lesion regions based on the corresponding attention matrix from different scales of the input image, respectively. The COLM is used for locating the main lesion object in the image at coarse-grained level, while the LPPM proposes lesion details in the image at medium-grained level. Once an attended region is located, we segment the region and zoom in it to the raw image size. The located regions can be employed to generate a series of highly reliable lesion features. As a whole, the CALP-CNN takes advantage of ensemble learning to integrate the features from three scales for final identification. Moreover, the CALP-CNN combines an intra-scale cross-entropy loss and an inter-scale pairwise ranking loss to ensure rapid convergence.

#### 2.2.1 Class response map

**Figure 3:**
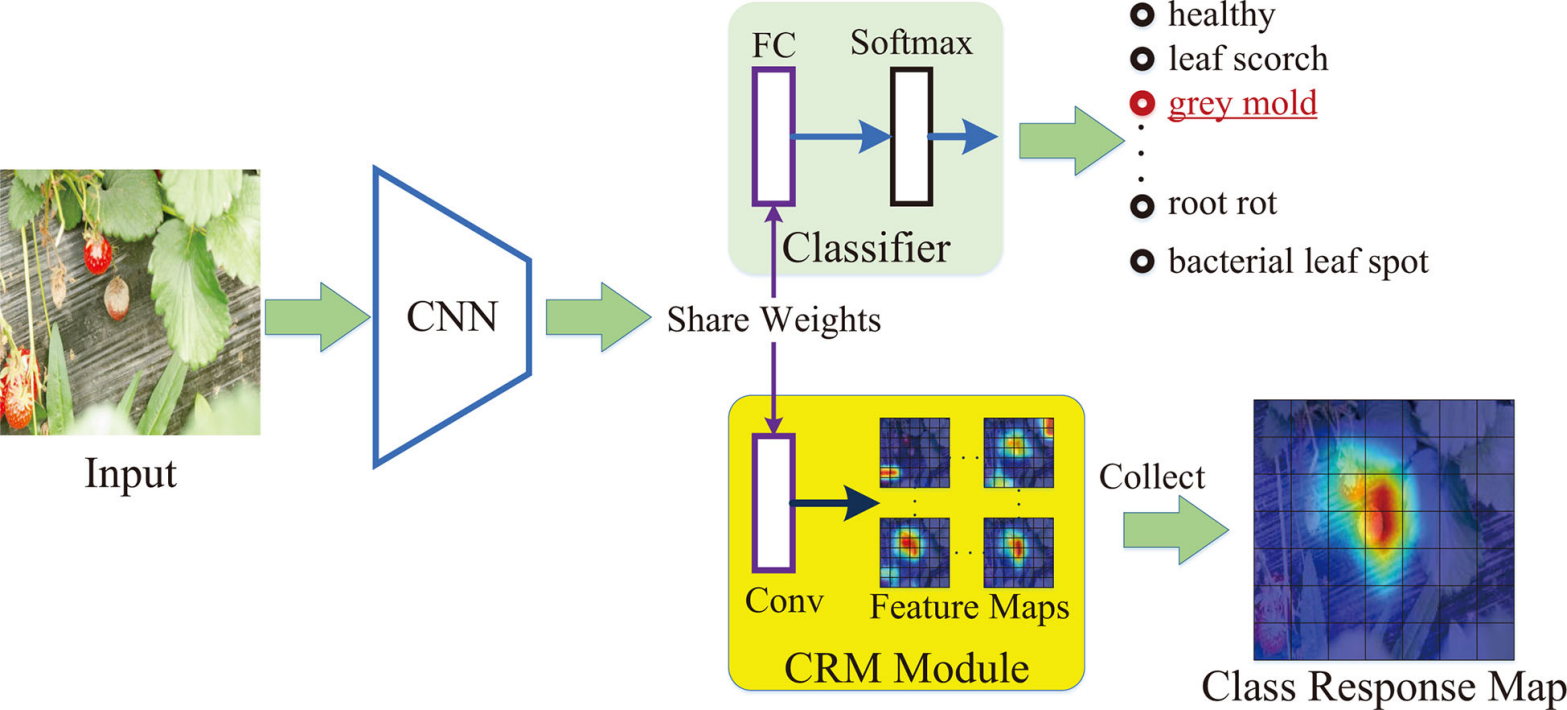
The generation process of class response map.

A series of class activation maps can be generated by the product of CNN feature maps with their corresponding class scores. The studies of (Zhou et al., 2016; Ding et al., 2019) have proved that the class-related information in the class activation maps is effective for locating discriminative regions in an image. In this paper, we obtain discriminative information of lesions based on the class activation maps and construct a class response map (also denoted as class attention matrix) to localize the objects of interest. **Figure 3** shows the generation process of a class response map.

First, a pre-trained CNN backbone is applied to extract the feature maps of a 3-channel image *I*∈*R* ^3×*H*×*W*^ , where the *H × W* is the spatial size of the image. The extracted feature maps are represented as *S*∈*R* ^*C*×*H*_*f*_^×*W*_*f*_^^,where *C* is the channel number and ^*Hf × Wf*^ is the spatial size of the feature maps. Second, the feature maps *S* are fed forward to a classifier consisting of a fully connected (FC) layer and a softmax layer. A vector p∈R^{N_c}.(*N_C_* is the pre-set category number of the strawberry diseases) can be computed by the classifier as the predicted probability score of each disease. In addition, the weights of the FC layer are denoted as *w*_*fc*_∈*R* ^*C*×*N*_*c*_^ . Third, a CRM module is designed to generate the class-related features maps. It establishes a new convolutional layer with the weight of the *w_fc_* (i.e., the formed convolutional layer achieves the same weights as the FC layer). Therefore, it possesses a strong ability to extract class-related features. Based on the constructed convolutional layer, a set of class-related feature maps *Q*={*Q*_*i*_}(*Q*_*i*_∈*R* ^*Hf*×*Wf*^,*i*=1,…,*N*_*c*_) can be generated from the extracted *S*. The *Q_i_* represents the *i*-th channel. The features of the *Q_i_* are most relevant to category *i*. In the training process, the CALP-CNN applies the ground truth label to select the most class-related feature map of the convolutional layer as the class response map. That is to say, if the image is annotated as category *c*, the class response map is *Q_c_*. In the testing process, there is no ground truth label of the input image. Follow as (Ding et al., 2019), the CALP-CNN adopts the entropy of the top 5 predicted probabilities to evaluate the lesion information in their corresponding class-related maps. Let $\hat{p}\in R^{5}$ be the subset of *p* for top 5 predicted class probabilities. We compute the entropy as


(1) $$H=-\sum_{i=1}^{5}p_{i}\cdot logp_{i},\;p_{i}\in \hat{p}$$

and construct the class response map *Q_c_* based on the following strategy,


(2) $$Q_{c}=\{\begin{array}{c} \hat{M_{1}},\;if\;H\leq \epsilon \\ \sum_{i=1}^{5}\hat{M_{i}},\;otherwise \end{array}$$

where $\hat{M}\in R^{5\times H_{f}\times W_{f}}$ is the class-related feature maps correspond to $\hat{p}$ and ε is a threshold (empirically set to 0.2).

#### 2.2.2 Class object location module (COLM)

**Figure 4:**
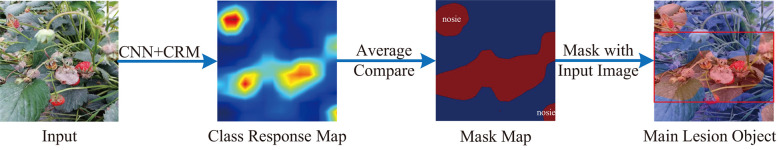
The pipeline of COLM. A class response map is generated from a CRM module. The pixels in the class response map are compared to their average value to generate a mask map. Some non-lesion areas are activated by the complex background in the mask map.

In most cases, the CNN backbone could extract many irrelevant and noisy features that are adverse to disease identification, especially for a complex background (Barbedo, 2018). To cope with this issue, we design the COLM to locate the main lesion object and discard the irrelevant background region. This module is inspired by the discriminative region location methods of the fine-grained image classification and retrieval domain (Wei et al., 2017; Ding et al., 2019; Zhang et al., 2021). The pipeline of COLM is shown as **Figure 4**

The class response map *Q_c_* is resized to the same size as the input image *I* by a bilinear interpolation algorithm. The interpolation result is denoted as $Q_{c}^{\prime }\in R^{H\times W}$ . Ding et al. have concluded that the larger value in the class response map, the more related of the corresponding pixel to the class (Ding et al., 2019). In most cases, we have no prior knowledge about the location of the lesion objects since most crop disease datasets only have image-level supervision.


(3) $$\bar{q}=\;\frac{\sum_{i=1}^{H}\sum_{j=1}^{W}Q_{c}^{\prime }(i,j)}{H\times W}$$

Then, a mask map *M* can be generated according to Eq.4.


(4) $$M(i,j)\;=\left\{ \begin{array}{l} 1,\;if\;Q_{c}^{\prime }(i,j)>\bar{q}\\ \;0,\;otherwise \end{array}\right.$$

As shown in **Figure 4**, the object regions are marked red in the mask map. We can observe some noisy regions (the top-left and bottom-right) in the mask. In fact, the noisy regions could be non-lesion parts, whereas they are activated by the complex background. Fortunately, the sizes of the noisy regions are typically smaller than the main lesion object. Flood-fill algorithm is a common method to connect neighboring and related elements of a matrix. In this paper, we apply it to test the connectivity of all the points in *M* and find out the largest connected area. The largest connected area is the location of the main lesion object. The minimum enclosing rectangle of the largest connected area is denoted as *M*. We adopt the top-left point (*x_tl_, y_tl_*) and bottom-right point (*x_br_, y_br_*) to represent the location of *M = M* [*x_tl_:x_br_,y_tl_:y_br_*]. With the interpolation algorithm, the pixels in the mask map *M* are one-to-one corresponding to the pixels in the input image *I*. Therefore, the location of *M* can be used to extract the main lesion object and discard the noisy background in *I*. As a result, the main lesion object *I_obj_* is computed as:


(5) $$I_{obj}=I[x_{tl},x_{br},y_{tl}:y_{br}]$$

Based on the ablation experiments in section 4.2, the COLM module can effectively improve the classification accuracy.

#### 2.2.3 Lesion part proposal module (LPPM)

Identifying similar diseases in the field is another critical problem for strawberry cultivation, especially for those diseases which have homologous backgrounds and subtle inter-class differences (e.g., the diseases at the early stage and the diseases occurring in the same part). Strengthening the differences between diseases is the key approach to address this issue (Cruz et al., 2019). The similar disease identification is in accord with the characteristics of the fine-grained image recognition (FGIR) (Zheng et al., 2017). The studies of FGIR have concluded that the discriminative features always lie in the details (Fu et al., 2017; Recasens et al., 2018; Ding et al., 2019; Zhang et al., 2021). Hence, we present the LPPM to localize the distinguishing lesion features in the details. The design idea of this module is derived from the region proposal algorithm (RPA) (Ren et al., 2015). The RPA is an effective method to propose candidate regions for object detection. The candidate region is called anchor in object detection. Nevertheless, the RPA requires an additional bounding box to annotate the location of the object. The bounding box annotation process is labor-intensive and subjective. Here, we take the average value of all pixels in the anchor as a confidence of whether the region in the anchor is a lesion detail. In this way, the RPA can be generalized to identify detailed lesions in the images without bounding box annotations.

**Figure 5:**
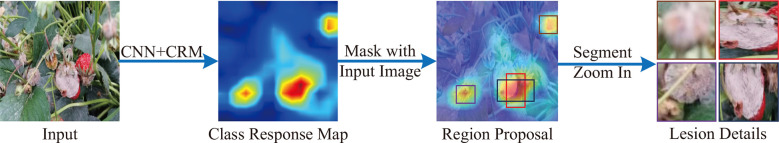
The pipeline of LPPM. First, a class response map is generated from a CRM module. Second, the RPA is applied to proposal candidate lesion regions from the class response map. Third, a non-maximum suppression is utilized to pick out the top-*N* lesions.

The pipeline of LPPM is shown as **Figure 5**. The LPPM takes the output (i.e., class response map) of a CRM module as input. We denote it as *M*_*c*_∈*R* ^*Hf*×*Wf*^ . First, the LPPM propose the coordinates of the anchors on *M_c_*. By default, we use 3 aspect ratios (1:1, 2:1, 1:2) and 1 scale (H*_f_*/2), yielding *k*=3 anchors at each pixel of *M_c_*. The total number of generated anchors is k × H*_f_* × W*_f_*. Each anchor is an eligible candidate for the lesion detail. The coordinates of the anchors are denoted by their top-left point ${\mathrm{(x}}_{tl}^{\prime },y_{tl}^{\prime })$ and bottom-right point ${\mathrm{(x}}_{br}^{\prime },y_{br}^{\prime })$ Second, we calculate the average value of an anchor at *M_c_* as follows:


(6) $$\bar{a}=\frac{\sum_{i=x_{\mathrm{tl}}^{\prime }}^{x_{\mathrm{br}}^{\prime }}\sum_{j=y_{\mathrm{tl}}^{\prime }}^{y_{\mathrm{br}}^{\prime }}M_{c}\left(i,j\right)}{\left(x_{\mathrm{br}}^{\prime }-x_{\mathrm{tl}}^{\prime }\right)\times \left(y_{\mathrm{br}}^{\prime }-y_{\mathrm{tl}}^{\prime }\right)}$$

$\bar{a}$ is the confidence of the anchor to be a lesion detail region. A higher value of ā represents the higher probability of the anchor being a lesion detail. Third, we pick out the top-*N* anchors according to their confidence. In practice, the top-*N* anchors are adjacent and contain almost the same parts (Ren et al., 2015). For this reason, the directly selection of top-*N* anchors will cause information redundancy.

**Algorithm 1:** 

**Input:** The coordinate list of the anchors; The corresponding confidence list of the anchors; The IoU *threshold*, **Output:** The top-*N* anchor listCombined the confidence list and the coordinate list with an element as $\left[\bar{a},\;x_{tl},y_{tl},x_{br},y_{br}\right]$ . The result is a confidence_coordinate_list; confidence_coordinate_list ← Sort the combined list in descending order with the confidence $\bar{a}$ ;anchor_list ← Initialize an empty list of selected anchors; **while** *Length(anchor_list)< N and Length(confidence_coordinate_list)* > *0* **do** A←Pop out the first anchor element from the confidence_coordinate_list; **If** *anchor_list is empty* **then**Add *A* to the anchor_list;**else** Calculate the *IoU* between A and the other anchors in the anchor_list;**if** *IoU< threshold* **then** *IoU< threshold* Add *A* to the anchor_list;**return** *the anchor_list (is the top-N list);*

In this paper, we use the intersection over union (IoU) to indicate the redundant ratio of two anchors. The IoU between anchor *A*_2_ and anchor *A*_2_ is computed as:


(7) $$IoU=\frac{A_{1}\cap A_{2}}{A_{1}\cup A_{2}}$$

The IoU ratios between the anchor with the highest confidence and the other anchors are calculated. The scores of neighboring anchors will be suppressed when their IoU ratios are higher than the pre-set threshold. The threshold is set to 0.7 in this paper. The selection process of the top-*N* anchors is described in Algorithm 1 Note that *N* is a hyper-parameter which represents the defined number of lesion details. From the ablation experiments (see Section 4.2), the CALP-CNN achieves the best classification results when *N* is set to 5. Finally, we map the coordinate of the anchors in the top-*N* list to the input image *I* with the stride (s = H/H_*f*_) of the backbone network. The location of the lesion *I_detail_* is generated as:


(8) $$I_{\text{detail}}=\;I[s*x_{tl}:s*x_{br},s*x_{br}:s*y_{br}]$$

#### 2.2.4 Optimization strategy

The loss function of the proposed CALP-CNN is composed of two parts, including an intra-scale cross-entropy loss *L_cls_* and an inter-scale pairwise ranking loss *L_rank_*. The total loss function for an image *I* is defined as follows:


(9) $$L(I)=\;L_{cls}(I)+L_{rank}(I)$$

The *L_cls_* and *L_rank_* are expressed in Eq. 10 and Eq. 11, respectively.


(10) $$L_{cls}\left(I\right)=L_{cls}\left(Y^{r},Y^{*}\right)+L_{cls}\left(Y^{o},Y^{*}\right)+L_{cls}\left(Y^{c},Y^{*}\right)+\sum_{i=1}^{N}L_{cls}\left(Y^{d_{i}},Y^{*}\right)$$

where *Y^r^*, *Y°*, and *Y^d^* are the predicted label vectors from the raw, object and detail images. *Y^c^* is the predicted label vector using the concatenated features and Y^*^ is the ground truth label vector. *N* is the number of lesion details. *L_cls_* is the chief loss function, which is dominant in the parameter optimization of the CALP-CNN.


(11) $$L_{rank}\left(I\right)=L_{rank}\left(p^{r},p^{o}\right)+\sum_{i=1}^{N}L_{rank}\left(p^{o},p^{d_{i}}\right)$$

where *p^r^*, *p°* and *p^d^* denote the prediction probabilities from the raw, object and detail images, respectively. To be specific, the ranking loss of the probabilities *p^i^* and *p^j^* is defined as:


(12) $$L_{rank}(p^{i},p^{j})=max\{0,p^{i}-p^{j}+\delta \}$$

where δ is a constant (by default, δ=0.05). The ranking loss can force the object image to acquire higher predicted probabilities than the original image. Meanwhile, the detail images are forced to acquire higher predicted probabilities than the object image. In other words, the *L_rank_* takes a coarse prediction as reference and gradually compels the network toward more discriminative region by forcing the finer-scale images to achieve more confident predictions.

### 2.3 Evaluation metrics

In this paper, the *Accuracy*, *Precision*, *Recall*, and *F*1-*score* are adopted to evaluate the performance of the proposed CALP-CNN. The *Accuracy*, *Precision*, *Recall*, and *F*1-*score* of category *i* are defined as follows:


(13) $$Accuracy_{i}=\frac{TP_{i}+TN_{i}}{TP_{i}+FP_{i}+TN_{i}+FN_{i}}$$

(14) $$Precision_{i}=\frac{TP_{i}}{TP_{i}+FP_{i}}$$

(15) $$Recall_{i}=\frac{TP_{i}}{TP_{i}+FN_{i}}$$

(16) $$F1-score_{i}=\frac{2Precision_{i}\cdot Recall_{i}}{Precision_{i}+Recall_{i}}$$

where *TP_i_* and *TN_i_* denote the number of samples labeled as category *i* and non-category *i* that are correctly classified, respectively. *FP_i_* denotes the number of samples labeled as non-category *i* but classified as category *i*. *FN_i_* denotes the number of samples labeled as category *i* but classified as non-category *i*.

For a multi-class classification task, the overall *Accuracy*, *Precision*, *Recall*, and *F*1-*score* can be defined with the average of all the categories in their binary classification case. The formulas of the overall *Accuracy*, *Precision*, *Recall*, and *F*1-*score* are defined as follows:


(17) $$Accuracy=\frac{\sum_{i=0}^{N_{c}-1}Accuracy_{i}}{N_{c}}$$

(18) $$Precision=\frac{\sum_{i=0}^{N_{c}-1}Precision_{i}}{N_{c}}$$

(19) $$Recall=\frac{\sum_{i=0}^{N_{c}-1}Recall_{i}}{N_{c}}$$

(20) $$F1-score=\frac{\sum_{i=0}^{N_{c}-1}F1-score_{i}}{N_{c}}$$

where the *N_c_* represents the number of categories of strawberry diseases in the SCDD.

## 3 Experimental results and analysis

We conduct a series of experiments on the testing set of the SCDD to verify the effectiveness of the proposed CALP-CNN to identify strawberry diseases by filtering the complex background features and learning the discriminative features among similar diseases. The top-N of the anchors (lesion details) is set to 5 for the LPPM in our experiments.

**Baselines:** Because the CALP-CNN is an attention-based model and our SCDD only has image-level supervision, here we select six weakly-supervised fine-grained image recognition methods as baselines and compare their disease identification performance with the CALP-CNN method. The six baselines are described in detail as follows:

- MA-CNN (Zheng et al., 2017): Multi-attention convolutional neural network, which uses channel grouping to learn different part features.
- RA-CNN (Fu et al., 2017): Recurrent attention convolutional neural network, which recurrent learns the finer-scale features by an attention proposal network.
- MMAL-Net (Zhang et al., 2021): Multi-branch and multi-scale attention network, which utilizes a saliency map to locate the main object and propose discriminative parts.
- SSN (Recasens et al., 2018): A saliency-based sampling layer for a neural network that samples the raw image based on a saliency map with a non-uniform method.
- TASN (Zheng et al., 2019): Trilinear attention sampling network first uses a trilinear function to enhance saliency values, then samples the raw images with these enhanced values.
- S3N (Ding et al., 2019): Selective sparse sampling network, which captures diverse and fine-grained detail from the raw image based on a class response map with a selective sparse method.

All the baselines achieve state-of-the-art on their fine-grained datasets [e.g., CUB-200-2011 (Welinder et al., 2010), and FGVC Aircraft (Maji et al., 2013)].

**Implementation details:** The proposed CALP-CNN is implemented on the open-source package Pytorch (Paszke et al., 2019), which can flexibly implement various CNN-based models. A pre-trained ResNet-50 on the ImageNet dataset is used as the backbone for extracting the feature maps. For a fair comparison, all baselines are re-implemented with this backbone. We use the stochastic gradient descent (SGD) to optimize network parameters. All the models are trained for 60 epochs with a batch size of 16. The initial learning rate is set to 1e-3 and will be dropped by 10 at the 20-th and 40-th epoch. The momentum is set to 0.9 and the weight decay is set to 1e-4. The input images are preprocessed to size 224×224. All the experiments are performed on a dell T5820 computer workstation with NVIDIA GeForce RTX 3090 GPU and Intel Xeon W-2200 processor.

### 3.1 Classification results

We compare the performance of the proposed CALP-CNN with the baselines on the testing set of the SCDD. The classification results are shown in **Table 2**. The CALP-CNN achieves more accurate classification results on all metrics. The CALP-CNN significantly outperforms the backbone (ResNet-50) by 9.49% on the *F*1-*score*. The overall *F*1-*score* of the CALP-CNN is higher than the saliency-based models, for example, 9.03% improvement for SSN, 7.05% improvement for TASN, and 6.52% improvement for MMAL-Net. Additionally, the proposed CALP-CNN is also superior to the recurrent attention method (RA-CNN), the channel grouping attention method (MA-CNN), and the class attention method (S3N). Specifically, it improves 8.59%, 8.4% and 6.55% compared with RA-CNN, MA-CNN, and S3N on *F*1-*score*, respectively. Note that the improvement of our proposed model is contributed by the introduction of the COLM and LPPM. The COLM can filter the noisy background features, while the LPPM provides discriminative lesion details.

**Table 2:** The classification performance of different methods on the SCDD.

	Attention Mechanism	*F*1-*score*	*Accuracy*	*Precision*	*Recall*
ResNet-50 (He et al., 2016)	–	82.47	84.35	83.49	82.13
RA-CNN (Fu et al., 2017)	part attention	83.37	85.71	84.56	83.38
MA-CNN (Zheng et al., 2017)	channel attention	83.56	85.82	84.49	83.92
MMAL-Net (Zhang et al., 2021)	saliency attention	**85.44**	**87.11**	85.79	**85.47**
SSN (Recasens et al., 2018)	saliency attention	82.93	84.40	84.01	82.91
TASN (Zheng et al., 2019)	saliency attention	84.91	87.10	85.72	84.88
S3N (Ding et al., 2019)	class attention	85.41	86.70	**86.56**	84.72
CALP-CNN	class attention	**91.96**	**92.56**	**92.55**	**91.80**

The bold and underlined values indicate the highest and sub-optimal scores in the metric, respectively.

### 3.2 Ablation experiments

In this paper, four ablation experiments are conducted to investigate the role of 1) different network branches, 2) lesion location methods (saliency map vs. class response map), 3) the number of lesion details, and 4) the ranking loss on field disease identification accuracy. The experiments show that the CNN with three branches and five lesion details (top-5) achieves the best performance. The best model is equipped with the class response map for lesion location and the ranking loss for model optimization.

#### 3.2.1 Contribution of different branches

As shown in **Figure 2**, the CALP-CNN consists of three main branches, i.e., the raw branch (R-branch), the object branch (O-branch), and the (lesion) details branch (D-branch). In our experiments, we temporarily remove different branches to survey the contribution of each branch. The *F*1-*score* of the ablation experiments is recorded in **Table 3**. The following conclusions can be drawn: 1) The *F*1-*score* of the CALP-CNN with all branches (R+O+D) is 91.96%. It drops to 87.94% when omitting the O-branch. While it drops to 88.42% when the D-branch is removed. These results demonstrate that both the O-branch and the D-branch are capable of locating informative lesion regions. 2) The O-branch has the highest score (88.97%) among the three branches. It shows that the locating and segmenting operation of the class-related lesion object from the complex background can effectively eliminate the influence of the background on disease identification in the field. 3) The D-branch represents detailed information on lesions but does not yield the highest score among the three branches. It demonstrates that the discriminative lesion detail features are not all-inclusive for disease identification. Contextual information is also a key feature for disease identification. On the other hand, the D-branch could provide essential information to the other branches. The overall accuracy of the network features is improved from 83.92% to 87.94% in R+D branches setting and 87.08% to 91.21% in O+D branches setting, respectively. Furthermore, the D-branch can collect important lesion details for similar disease identification cases. 4) Note that the absence of the O-branch results in a bigger loss (4.02%, from 91.96% to 87.94%) than the D-branch (3.54%, from 91.96% to 88.42%), suggesting that removing the background features is critical for disease identification in the field. 5) The concatenated features of the three branches achieved the best performance. It indicates that the share of the object and the lesion detail features can enhance the lesion features and suppress the influence of background features. The disease surrounding context information of disease is preserved in the concatenated features.

**Table 3:** The contribution of each branch.

Experimental Setting	R-branch(%)	O-branch(%)	D-branch(%)	Concatenation(%)
R branch	82.47	–	–	82.47
R+O branches	82.66	88.97	–	88.42
R+D branches	83.92	–	83.01	87.94
O+D branches	–	87.08	84.37	91.21
R+O+D branches	82.44	88.12	86.05	91.96

#### 3.2.2 Role of different location methods

We re-implement the COLM and LPPM with saliency-based attention (Zhang et al., 2021) to locate the main object and the lesion details. The saliency map adopts a class-agnostic attention mechanism. Different from the saliency map, the class response map is a class-aware attention method. From **Table 4**, we can observe that the class-aware method has 5.57% higher scores than the class-agnostic method. It further demonstrates that the class-aware method can effectively localize class-related regions.

**Table 4:** Comparison between different location methods.

	*F*1-*score*(%)	Comments
saliency map	86.39	class-agnostic attention
class response map	91.96	class-aware attention

**Figure 6:**
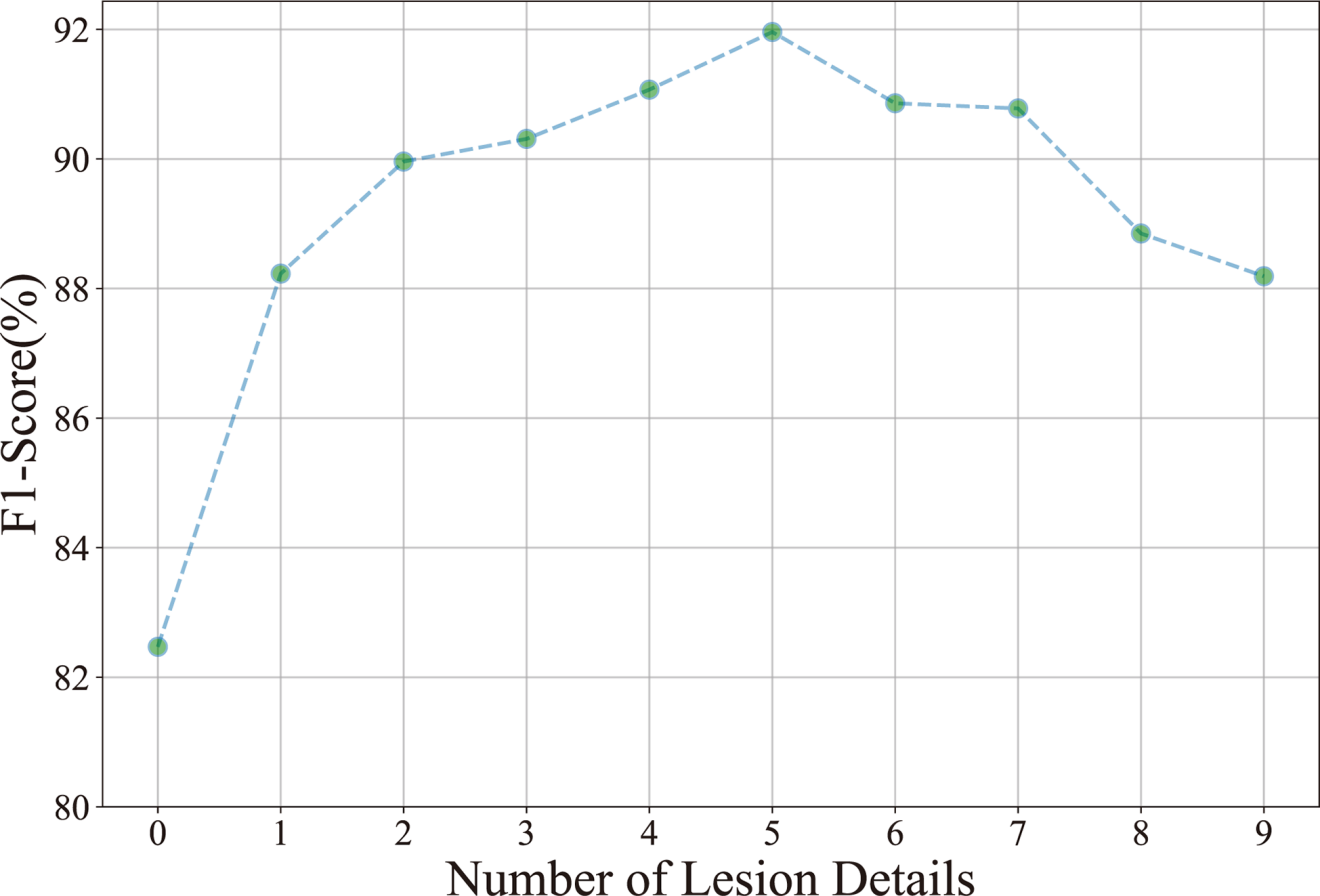
Relationship between the classification accuracy (*F*1-*score*) and the number of lesion details.

Number of lesion details: Ten experiments are performed to investigate the relationship between the classification result (*F*1-*score*) and the number of lesion details. As shown in **Figure 6**, the *F*1-*score* improves as the number of lesion details increases. However, the *F*1-*score* declines when the number of lesion details exceeds 5. It demonstrates that the disease classification performance is not positive to the number of lesion details. The underlying reason is that the contextual information is diluted in numerous detailed lesions.

#### 3.2.3 Effect of ranking loss

To explore the impact of the ranking loss on classification results, we remove the ranking loss and only retain the cross-entropy loss to optimize the parameters of the CALP-CNN model. The best *F*1-*score* in 60 epochs training is 91.30%, which is 0.66% lower than the original model. The introduction of ranking loss could assist the two modules (COLM and LPPM) in localizing more discriminative regions.

### 3.3 Results of similar diseases identification

**Figure 7:**
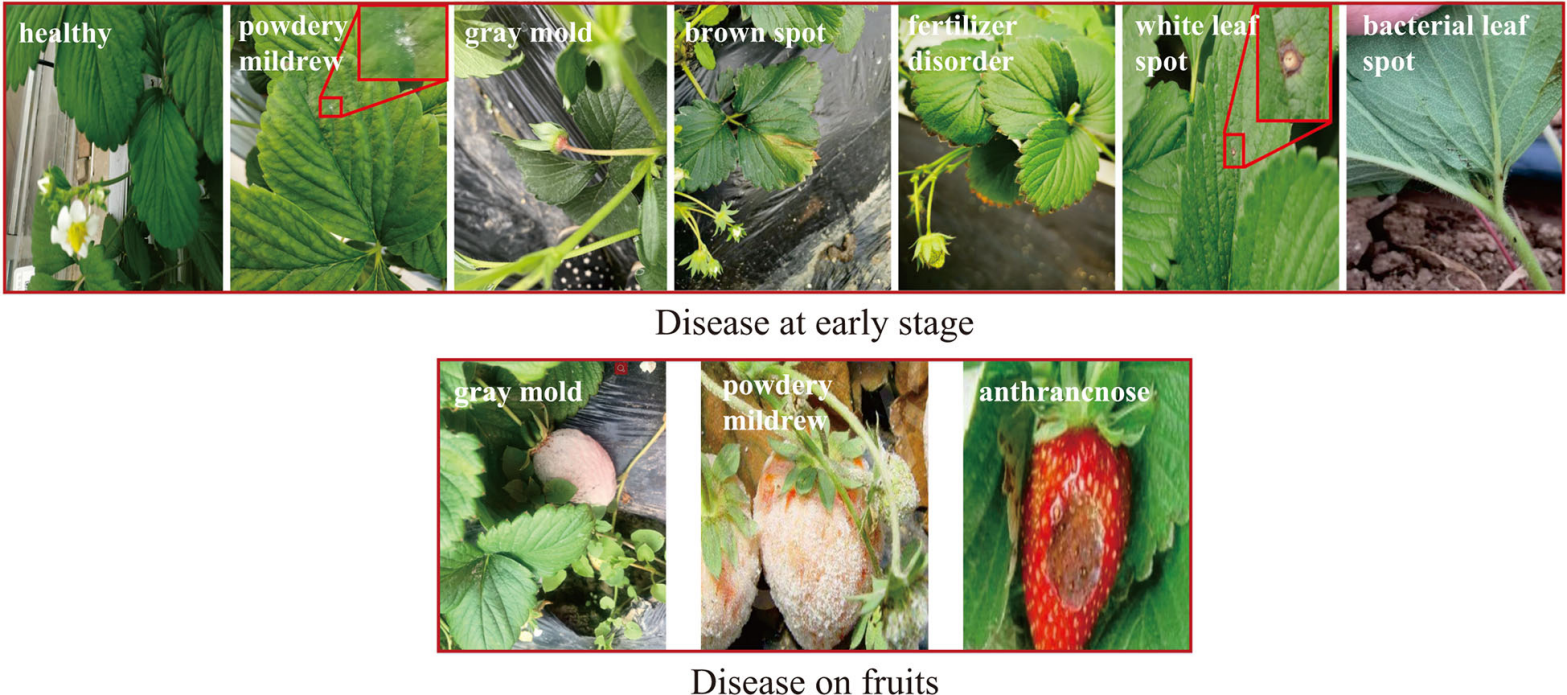
The examples of the similar diseases in the SCDD.

In practice, some of the diseases of strawberries perform similar visual appearance and contextual information, which could result in false identification among similar diseases. In order to evaluate the effectiveness of the proposed CALP-CNN for distinguishing these similar diseases, two kinds of similar strawberry diseases are chosen in the SCDD for experiments, including (1) the diseases at early stage, (2) the diseases occurring on fruits (e.g., gray mold, powdery mildew, anthracnose). We generate two sub-datasets corresponding to the two kinds of similar strawberry diseases. The disease samples from the two sub-datasets are shown in **Figure 7**.

The validation results of the trained CALP-CNN and the ResNet-50 on the two sub-datasets are recorded in **Table 5**. Both of the methods do not achieve the ideal identification performance. However, our CALP-CNN outperforms the ResNet-50 by 5.85% on disease at early stage dataset and 6.73% on disease on fruit dataset, respectively. Overall, the results suggest that the identification of similar strawberry diseases is challenging. While the discriminative lesion detail features provide helpful information to improve the identification performance.

**Table 5:** The performance of the CALP-CNN and the ResNet-50 on the similar disease datasets.

Dataset	Amount/Categories	ResNet-50	CALP-CNN
early stage	324/10	59.87	65.72
on fruit	79/3	69.30	76.03

### 3.4 Qualitative evaluation of lesion localization performance

**Figure 8:**
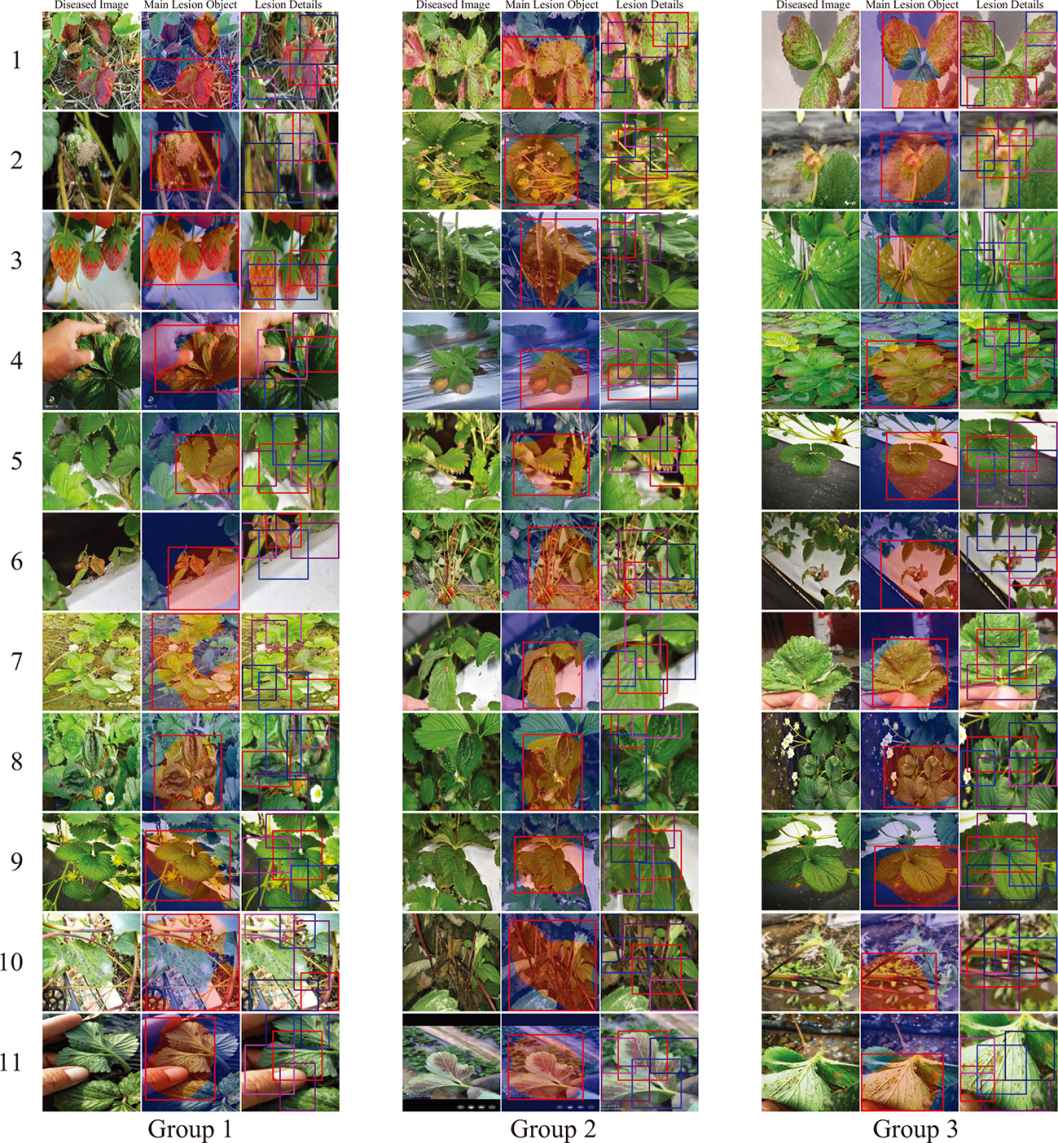
Identified main lesion object and lesion details. For each disease, we randomly select three samples from the testing set. The first column of each sample is the diseased image, and the subsequent two columns are the location results of the main lesion object and lesion details. The labels of the diseases are consistent with the **Table 1**.

Because most of the strawberry datasets (including the SCDD) are image-level annotations. It is difficult to quantitatively evaluate the location accuracy of the main lesion object and the lesion details at the pixel-level. Here, we follow the study of (Wei et al., 2017) to conduct a qualitative evaluation to evaluate the accuracy of the main lesion object and lesion detail detection. We randomly pick out 3 groups of diseased images from the testing set for each strawberry disease and visualize the identification results of the lesions. The experimental results are shown in **Figure 8**. In **Figure 8**, the first column of each group is the input image, and the subsequent two columns are the location results of the main lesion object and lesion details of the image, respectively. Note that the images of lesion detail have been amplified to the same size as their input images. Based on the results of the main lesion objects, we can observe that the main lesion objects are all identified in the predicted bounding boxes of the COLM (group 1: 11/11, group 2: 11/11, group 3: 11/11). Furthermore, the predicted boxes contain contextual information by persevering the local background of the main lesion objects. In addition, most lesion details of the diseases can also be predicted by the LPPM (group 1: 54/55, group 2: 52/55, group 3: 55/55). In our experiments, the false predicted lesion areas occur in the images which have only one lesion area and the size of the lesion is relatively small (e.g., line 7, column 3 of group 2).

## 4 Discussions and conclusions

Existing methods for crop disease identification in the field are not sufficiently accurate because of their poor ability to eliminate the interference from the background and extract discriminative features among similar diseases. Detecting and segmenting the lesion region from the disease image is a simple yet effective way to reduce the influence of the complex background. Meanwhile, learning discriminative features from the lesion details is beneficial for the identification of similar diseases. The CNN-based semantic segmentation methods can effectively segment the lesion regions from the complex background. Hence, recent studies use semantic segmentation networks to segment lesion regions from the background as the first step of their models (Hu et al., 2021; Wang et al., 2021a). The segmentation performance of the networks highly relies on the amount of pixel-level annotated data. The pixel-level annotation is time-consuming, laborious and expensive, which restricts the applications of CNN-based segmentation methods. Besides, many studies have shown that the CNNs can localize discriminative regions from the input image (Selvaraju et al., 2017; Dabkowski and Gal, 2017; Wei et al., 2017; Ding et al., 2019). However, not all the located regions are useful for disease identification. The regions, which are activated by the complicated background, are adverse for disease identification (Barbedo, 2018). Therefore, it is necessary to filter out the most useful region from the located regions. The identification of similar diseases is also a challenging task. Because the discriminative details between the similar diseases are too subtle to be well-represented by the CNNs. Data augmentation technologies can increase the differences among similar diseases. Nevertheless, the increment is not obvious (Cruz et al., 2019). In addition, a suitable augmentation strategy is not straightforward and requires trial and error. Hence, data augmentation technologies are not an appropriate solution for similar disease identification. Fortunately, there are many similarities between crop similar disease identification and FGIR. The FGIR focuses on how to effectively represent the discriminative features between the subordinate classes (Ding et al., 2019). Therefore, the discriminative region localization and feature representation methods in FGIR can be extended to crop similar disease identification.

In this paper, we cite the field strawberry disease identification as our study object and explore innovative methods to address the challenges caused by the complex background and similar diseases. First, we enhance the ability of the CNN backbone to localize discriminative regions through a new class-attention-based mechanism (i.e., class response map). Second, we construct the COLM based on the flood-fill algorithm to filter out the most useful lesion region from the complex background. Third, we raise a new lesion part proposal method (i.e., the LPPM) to propose the discriminative lesion details based on the RPA. The COLM and LPPM are connected in series to form a Class-Attention-based Lesion Proposal Convolutional Neural Network (CALP-CNN), which can simultaneously address the challenges caused by complex background and similar diseases in field disease identification.

A series of experiments are conducted on the constructed field strawberry common disease dataset to testify the effectiveness of the CALP-CNN in eliminating the interference from the complicated background and distinguishing similar strawberry diseases. The classification result on *F*1-*score* reaches 91.96%, which is greatly higher than other methods, showing that the proposed model outperforms other state-of-the-art methods in the view of field strawberry disease identification. In addition, the ablation results on *F*1-*score* drop to 87.94% and 88.42%, respectively, when the COLM and LPPM branches in the CALP-CNN are removed. It indicates that both background feature elimination and discriminative lesion detail feature representation are indispensable for field disease identification.

## Data availability statement

The original contributions presented in the study are included in the article/supplementary material. Further inquiries can be directed to the corresponding authors.

## Author contributions

XH, RW, JD, and TX conceived the idea and designed the network. XH, LJ, and YH contributed to collecting the dataset. XH wrote the code, validated the method, and wrote the paper. TX, JD and LJ revised the paper. All authors contributed to the article and approved the submitted version.
